# Development of digital diagnostic templates by cluster analysis based on 2249 lateral cephalograms of Chinese Han population

**DOI:** 10.1186/s13005-022-00309-2

**Published:** 2022-02-14

**Authors:** Hong Su, Wenhsuan Lu, Jingjing Deng, Gui Chen, Ruoping Jiang, Yan Wei, Xiaoyun Zhang, Tianmin Xu, Bing Han

**Affiliations:** 1 Department of Orthodontics, Cranial-Facial Growth and Development Center, Peking University School and Hospital of Stomatology, 22 Zhongguancun South Avenue, Haidian District, Beijing, 100081 People’s Republic of China; 2 First Clinical Division, Peking University School and Hospital of Stomatology, 37A Xishiku Street, Xicheng District, Beijing, 100034 People’s Republic of China; 3 National Center of Stomatology & National Clinical Research Center for Oral Diseases & National Engineering Research Center of Oral Biomaterials and Digital Medical Devices & Research Center of Engineering and Technology for Computerized Dentistry Ministry of Health & NMPA Key Laboratory for Dental Materials, 22 Zhongguancun South Avenue, Haidian District, Beijing, 100081 People’s Republic of China; 4 Department of Geriatric Dentistry, Peking University School and Hospital of Stomatology, 22 Zhongguancun South Avenue, Haidian District, Beijing, 100081 People’s Republic of China

**Keywords:** Dentofacial deformity, Cluster analysis, Discriminant analysis, Cephalometric morphology template, Diagnosis

## Abstract

**Background:**

To establish the digital diagnostic templates by cluster analysis based on a set of cephalometric films and evaluate the outcome of the different treatment methods in the patients affiliated to the same cephalometric morphology template (CMT). These templates could be used for the automatic diagnosis of dentofacial deformities and prediction of treatment outcomes in the future.

**Methods:**

In this study, we assessed the coordinates of 60 different landmarks on the cephalograms of 2249 patients (14.35 ± 4.99 years, range from 7 to 62) with dentofacial deformities. The cephalometric data were subjected to dentist for clustering without a priori pattern definitions to generate biologically informative CMTs. Three templates were selected to evaluate the treatment outcome of patients affiliated to the same CMT.

**Results:**

The cluster analysis yielded 21 distinct groups. The total discriminant accuracy was 89.1%, while the cross-validation accuracy was 85.0%, showing that the clusters were robust. All CMTs were automatically created and drawn using a computer, based on the average coordinates of each cluster. Individuals affiliated to the same CMT showed similar dentofacial features. We also evaluated differences in the outcomes of patients affiliated to the same CMT.

**Conclusions:**

Our results demonstrated the utility of clustering methods for grouping dentofacial deformities with similar dentofacial features. Clustering methods can be used to evaluate the differences in the outcomes of patients affiliated to the same CMT, which has good clinical application value.

**Supplementary Information:**

The online version contains supplementary material available at 10.1186/s13005-022-00309-2.

## Background

Dentofacial deformities are a heterogenous group of disorders that affect numerous people worldwide. Orthodontists and orthognathic surgeons require a comprehensive understanding of the cranio-dento-facial tissues of each patient to arrive at an accurate diagnosis and devise appropriate treatment plans. Since the X-ray technique [1] was introduced into the orthodontic field, cephalograms have become one of the most important tools to understand dentofacial structures, predict growth, make diagnoses and evaluate treatments. Before computerization, manual cephalometric analysis was mainly used to assist orthodontists in analyzing the mechanisms underlying malocclusions. The existing analytic methods of cephalometry are mostly different combinations of line distances and angle measurements [2–4], which are used to diagnose deformities on the basis of comparisons with reference values. However, even more data of cephalometrics are unable to represent the overall craniofacial morphology very well and directly deduce the overall dentofacial morphology from different combinations of partial comparisons. In addition, the use of routine cephalometrics is time-consuming, the operator needs to consider all the possible cross-over interpretation of cephalometric value. To overcome this problem, graphic template analysis based on average dentofacial morphologies was developed to enable comprehensive morphological diagnoses and give the operator the opportunity to be more efficient with less-consuming time and less effort.

Manual templates formed from an expanding cephalogram database of growing individuals have been used to evaluate the overall dentofacial morphology of patients [5, 6] and even to predict growth [7]. Likewise, cephalograms of patients with malocclusions have been age-matched with templates prepared using cephalograms of individuals with normal occlusion from birth to 18 years of age, so as to enable diagnoses and explore the mechanisms of malocclusion [8]. These studies showed that template analysis makes it much easier to judge the contour features of different growth patterns and is more intuitive than traditional cephalometric measurements. Therefore, we consider that digital, automated template matching might be the key to realize fully automatic, computed diagnoses of dentofacial deformities and predict treatment outcomes.

The first step in establishing applicable digital templates is to develop reasonable sample classifications. Current classifications of malocclusion are mostly based on dental relationships such as Angle’s classification or on certain skeletal characteristics such as the ANB angle or MP/SN angle [3, 4]. Although a single value can help to judge a certain dentofacial feature, it cannot accurately represent the overall dentofacial morphology, since different orthodontists prefer different cephalometric indexes, the overall patient evaluation can vary. In recent years, a growing number of authors have started using mathematical methods such as geometry, statistics and computer analysis to assess the overall dentofacial morphology and explain similarities between samples [9–18]. One such method, cluster analysis, can group individuals in a given sample according to a certain feature and therefore provides a detailed description of the population while taking into account its intrinsic heterogeneity. This type of analysis has been garnering increasing attention in the dental field for classification purposes and epidemiological studies [19–23]. They have demonstrated that clustering the patterns of dentofacial deformities is beneficial for epidemiological diagnosis, treatment evaluation, and outcome prediction. Some authors have tried to cluster certain types of malocclusions into subclasses with typical dentofacial characteristics [20]. In addition to classification purposes and epidemiological studies, cluster analysis has also been used for more practical aspects of orthodontics [18]. Some authors have used it to identify anatomic characteristics that may influence bonding in the straight-wire technique. However, because most of these studies were partial and based on relatively small samples, their results pertain to only specific categories of certain types of malocclusions, and are limited in their ability to classify all types of dentofacial deformities.

This study aimed to cluster the cranio-dento-facial deformities of 2249 patients without a priori pattern definitions into biologically informative cephalometric morphology templates (CMTs) based on cephalogram coordinates and to explore the utility of the resultant templates for diagnosis and treatment evaluation. In this way, we hoped to help provide a novel method to evaluate and predict treatment outcomes.

## Materials and methods

### Sample recruitment and data collection

The sample used in this study was recruited through a database comprising > 11,000 patients who had malocclusion and had finished orthodontic treatment between 1997 and 2005 at the Peking University School and Hospital of Stomatology (PKUSS; Beijing, China). The study protocol was approved by the ethics committee of Peking University School of Stomatology (PKUSSIRB-201626016), got approval to dispense with informed consent, and adhered to the tenets of the Declaration of Helsinki. The study has been registered with the Chinese Clinical Trial Registration (ChiCTR1800017694).

The inclusion criteria were as follows: Chinese Han population; no hereditary diseases; undamaged lateral cephalograms taken before and after the treatments were available; and all cephalograms were obtained using Orthopantomograph OC100 (Instrumentarium Dental, Nahkelantie, Finland.) Data on several covariates were collected. The clinical records were used to gather data on sex, age at first visit, birth year, Angle’s classification, treatment methods, treatment design, and treatment duration. A total of 2249 patients in the database who met the inclusion criteria formed the study cohort. Among the 2249 study patients, there were 758 males (33.7%) and 1491 females (66.3%), with a mean age of 14.35 ± 4.99 years at the beginning of treatment (range from 7 to 62). Angle class I malocclusions were the most common (945 patients; 42.0%), followed by class II (872 patients; 38.8%) and class III malocclusions (432 patients; 19.2%).

### Landmark identification

Lateral cephalograms were provided by the radiology department of PKUSS. To control magnification, all head films were taken with the same cephalostat. All traditional films were calibrated for length scales and then scanned into digital image files (.tiff). The magnification produced from the projection and scanning procedures was calculated and corrected by the computer.

**Fig. 1:**
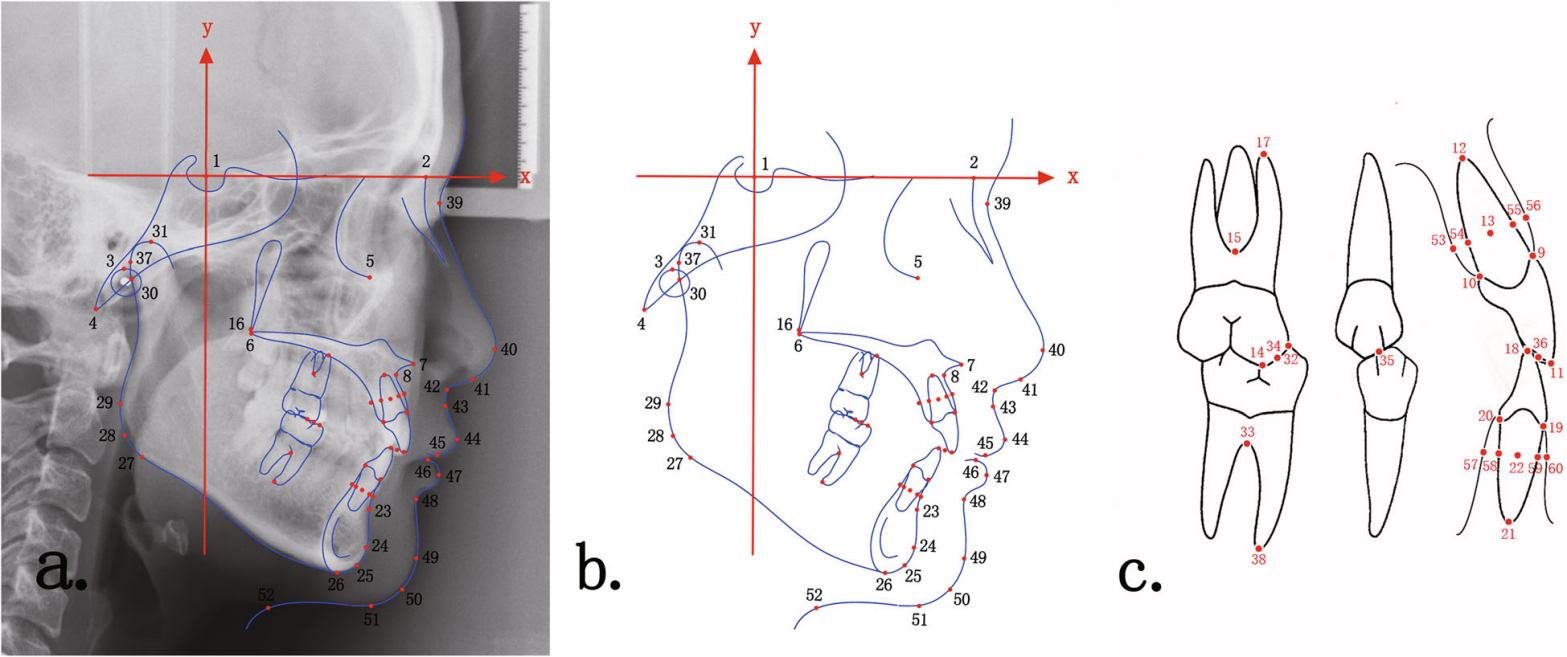
Cephalometric landmarks (schematic) of craniofacial hard and soft tissues. 1: sella (S); 2: nasion (N); 3: porion (P); 4: basion (Ba); 5: orbitale (Or); 6: posterior nasal spine (PNS); 7: anterior nasal spine (ANS); 8: subspinale (A); 9: superior prosthion (SPr); 10: palatal superior prosthion (Spr(p)); 11: edge of upper incisor (UIE); 12: apex of upper incisor (UIA); 13: resistance of upper incisor (UIR); 14: mesial buccal cusp of upper first molar (UMC); 15: resistance of upper first molar (UMR); 16: pterygomaxillary fissure (Ptm); 17: mesial buccal apex of upper first molar (UMA); 18: edge of lower incisor (LIE); 19: infradentale (Id); 20: lingual infradentale (Id(l)); 21: apex of lower incisor (LIA); 22: resistance of lower incisor (LIR); 23: supramental (B); 24: pogonion (Pg); 25: gnathion (Gn); 26: menton (Me); 27: tangent gonion (Tgo); 28: gonion (Go); 29: posterior gonion (Gop); 30: articulare (Ar); 31: condylion (Co); 32: mesial cusp of lower first molar (LMC); 33: resistance of lower first molar (LMR); 34: midpoint of UMC and LMC (Interdental-M); 35: midpoint of upper and lower second bicuspids (Interdental-B); 36: midpoint of UIE and LIE (Interdental-I); 37: posterior condylion (Cop); 38: mesial apex of lower first molar (LMA); 39: nasion of soft tissue (Ns); 40: pronasale (Prn); 41: columella (Cm); 42: subnasale (Sn); 43: subspinale of soft tissue (As); 44: upper lip (UL); 45: stomion superius (Stoms); 46: stomion inferius (Stomi); 47: lower lip (LL); 48: supramental of soft tissue (Bs); 49: pogonion of soft tissue (Pos); 50: gnathion of soft tissue (Gns); 51: menton of soft tissue (Mes); 52: cervical point (C); 53: posterior-resistance-on-bone of upper incisor (URP-p); 54: posterior-resistance-on-root of upper incisor (URP); 55: anterior-resistance-on-root of upper incisor (URA); 56: anterior-resistance-on-bone of upper incisor (URA-a); 57: posterior-resistance-on-bone of lower incisor (LRP-p); 58: posterior-resistance-on-root of lower incisor (LRP); 59: anterior-resistance-on-root of lower incisor (LRA); 60: anterior-resistance-on-bone of lower incisor (LRA-a)

Cephalometric landmarks were located by three residents who were blinded to the goals of our study and professionally trained in the definition of each point and the method of calibrating points by the rules of standardized cephalometrics each orthodontic resident had to master. We learned the method from Baumrind and Frantz’s study and obtain the personalized definitions of each landmark positions [24]. Outliers, if present, were mostly caused by inadvertent clicking on the screen, could be automatically detected with CIS (Cephalometric Information System, Peking University, Beijing, China) and were checked by the same individual. Regarding the calibration, after the residents located the cephalometric landmarks, we obtained three points of a landmark. Among the three points, if the maximum distance between two points was greater than two times the minimum distance between two points, the landmark was recalibrated. The average of three landmarks was used for subsequent calculations. In all, 46 hard-tissue landmarks and 14 soft-tissue landmarks were located. All the cephalometric landmarks have been shown and explained in a schematic plot (Fig. 1).

### Measurement and output

A commonly used coordinate system was employed in this study. We set the sella point (S) as the origin and the anterior cranial base plane (SN) as the x-axis to establish a Cartesian coordinate system (Fig. 1). All the coordinates of each located landmark were outputted to form a coordinate database.

Although the primary objective of the present study was to form a digital template series, we also automatically calculated and analyzed 135 angular and linear measurements based on the coordinates by means of CIS. All measurements were listed in Table 1. The measurement values were not used as variable ranges in the cluster analysis, but were used to describe features that helped clinicians understand and apply the diagnostic templates.

**Table 1:** All the cephalometric measurements

SNA	SN/OP2	A-NPg	Ns-Pos
SNB	FH/OP1	U1/NA	Ns-Sn
ANB	FH/OP2	UIE-NA	NLA(Cm-Sn-UL)
SNPg	OP1/MP	U1/PP	Ns-Mes
NSBa	OP2/MP	U1/FH	Stoms-Stomi
NSAr	OP2/PP	ANPr	Ns-Sn
FH/NA	OP1/PP	UIE-AP	Sn-Stoms
SN/FH	OPu/PP	UIE-PP	Sn-Mes
S-N	OPl/MP	UMC-PP	Stomi-Bs
S-Ba	L1/OP1	U1/SN	Stomi-Mes
Ba-N	LI/OPL	L1/NB	Stoms-UIE
A-NFH	LI/OP2	L1/MP	UIR-UMR
Ptm-S(FH)	UI/OPu	LI/FH	UIR-UMR(PP)
PNS-ANS(FH)	UI/OP1	CL/MP	UIE-UMC
PNS-A(FH)	UI/OP2	LIE-AP	UIA-UMA
Ptm-A(FH)	LM/OPL	LIE-MP	UIA-UMA(PP)
FH/NB	LM/OP1	LIE-NB	LIR-LMR
NGoGn	LM/OP2	LMC-MP	LIR-LMR(MP)
SGoN	UM/OP1	U1/L1	LIE-LMC
MP/SN	UM/OP2	UM/LM	LIA-LMA
MP/FH	NsPos/FH	overjet	LIA-LMA(MP)
NSGn	S-Ns-Sn	overbite	UM/PP
SArGo	S-Ns-Bs	UMC-LMC(OP1)	LM/PP
B-NFH	Sn-Ns-Bs	UMC-LMC(OP2)	Ptm/UMC(FH)
SL	S-Ns-Pos	SN/OP1	
SE	AsUL_BsLL	Co-Gn	Prn-H
Co-S(FH)	AsUL-FH	Go-Gn	Sn-H
N-Me	BsLL-FH	Pg-NB	Bs-H
S-Go	PosBs-FH	ANPg	LL-H
S-Go/N-Me	Ns-Prn-Pos	NA/PA	UL-EP
MP/ArGoP	Z angle	MP/PP	LL-EP
Co-Pg(MP)	H angle	Wits	UL-SnPos
ArGoGn	LL-Bs-Pos	A-B(FH)	LL-SnPos
Co-B	Sn-Gns-C	Co-Pg	Prn-SnFH

### Statistical analysis

Data were analyzed using SPSS version 16.0 (SPSS, Chicago, IL, USA). After clustering, the same coordinates were used as variables to perform discriminant analysis of all the clustering samples in order to obtain the discriminant equation. The derived discriminant equation should ensure the minimum error rate when the classification of a new sample is to be judged. The general form of the linear discriminant equation is as follows:

1 $$ Y=a1x1+a2x2+a3x3+\cdots \cdots + anxn, $$

where *Y* is the discriminant value, corresponding to CMTs classify. Variables reflecting characteristics of the sample are represented as *× 1, × 2, × 3 … xn,* corresponding to cephalometric measurements, and coefficients of the variables are represented as *a1, a2, a3 … an.*

After the discriminant equations were obtained, the coordinates of the original sample were substituted into the equations to calculate the overall accuracy of the discrimination. In addition, coordinates of different samples were also tested in a cross-validation analysis to verify the cross-discriminant accuracy.

The average coordinates of the samples corresponding to a particular category, as determined by the cluster analysis, reflected the mean morphology of that category. We selected some samples from certain subdivisions that are typically encountered in the clinic to determine differences in the effects of different treatment methods applied to the same deformity category. The Student *t*-test was used to compare the effects of the treatments. The level of statistical significance was set at *P* < 0.05.

## Results

### Cluster analysis

**Fig. 2:**
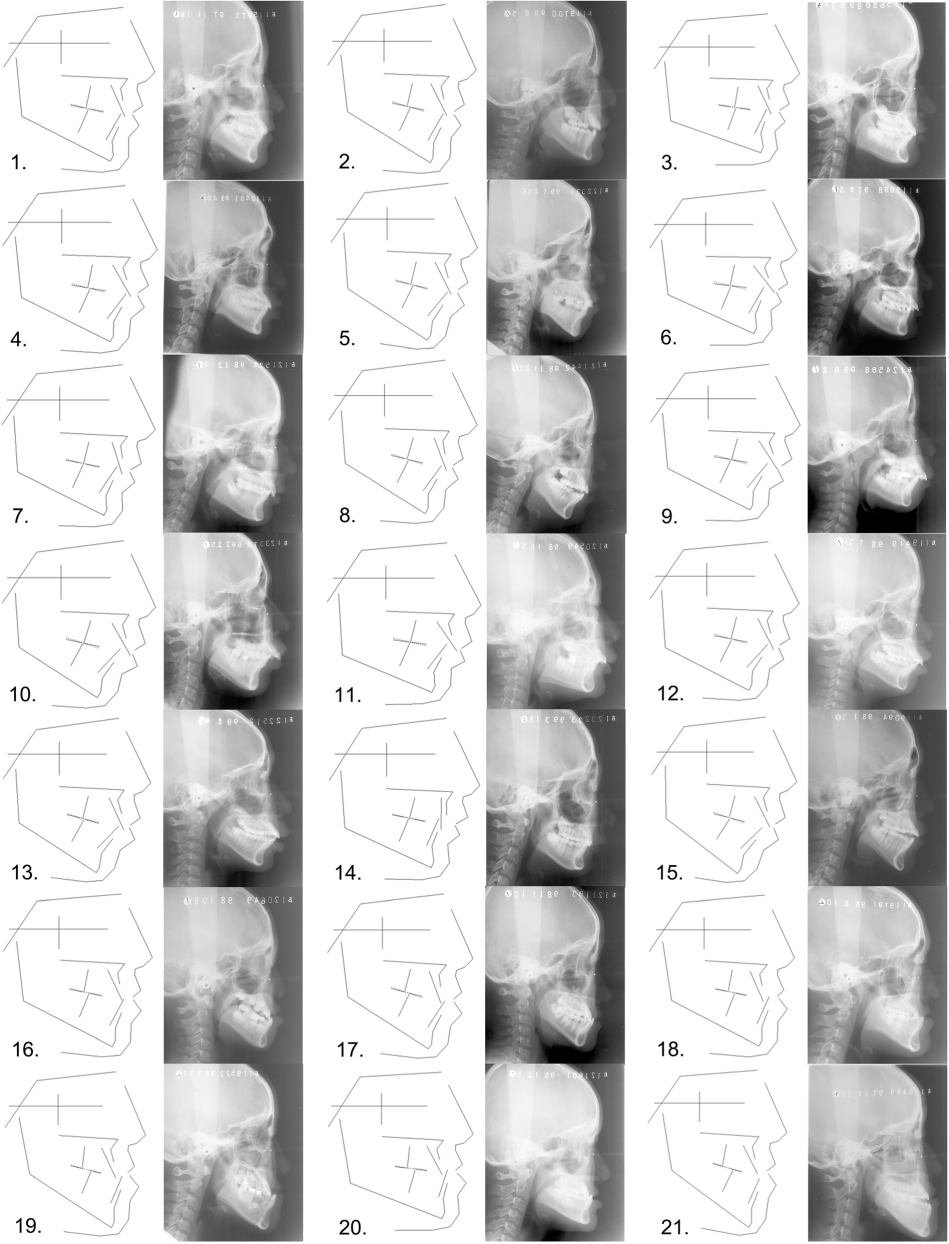
All the CMTs formed in this study and the corresponding typical cephalograms are showed and described referring to conventional cephalometric measurements. 1: skeletal I, mean angle, straight profile; 2: skeletal I, high angle, straight profile; 3: skeletal I, low angle, straight profile; 4: skeletal I, mean angle, bimaxillary protrusion; 5: skeletal I, high angle, bimaxillary protrusion; 6: skeletal I, deep overjet; 7: skeletal II, mean angle, retruded mandible; 8: skeletal II, high angle, retruded mandible; 9: skeletal II, mean angle, deep overjet; 10: skeletal II, high angle, deep overjet; 11: skeletal II, low angle, deep overjet; 12: skeletal II, mean angle, bimaxillary protrusion; 13: skeletal II, high angle, bimaxillary protrusion; 14: skeletal II, mean angle, lingually inclined incisors; 15: skeletal II, high angle, openbite; 16: skeletal III, mean angle, protruded mandible; 17: skeletal III, high angle, protruded mandible; 18: skeletal III, mean angle, retruded maxilla and protruded mandible; 19: skeletal III, high angle, retruded maxilla and protruded mandible; 20: skeletal III, low angle, retruded maxilla and protruded mandible; 21: skeletal III, high angle, openbite

The data of the 2249 patients were subjected to cluster analysis. After the exclusion of subclasses with sample sizes of less than 5, there remained a total of 21 subclasses. An average diagram of the craniofacial structures of each subclass was drawn based on the computed average coordinates. All 21 CMTs are shown in Fig. 2, and exhibit the mean structures of all the typical subclasses of dentofacial deformities. The initial analysis identified that the clusters (subclasses) could be described in terms of conventional cephalometric measurements. The distribution of the samples among the 21 CMTs is shown in Table 2. Table 3 is a summary table of CMT features.

**Table 2:** The distribution of the number of patients in the 21 CMTs

CMT type	sample	Percentage(%)	Male	Female	Mean age
CMT_1	190	8.5	48	142	14.4
CMT_2	132	5.9	41	91	13.9
CMT_3	53	2.4	18	35	16.5
CMT_4	249	11.1	93	156	13.7
CMT_5	178	7.9	60	118	13.6
CMT_6	80	3.6	21	59	14.5
CMT_7	97	4.3	26	71	14.5
CMT_8	68	3.0	6	62	13.9
CMT_9	155	6.9	64	91	12.9
CMT_10	102	4.5	39	69	14.0
CMT_11	44	2.0	24	20	14.4
CMT_12	251	11.2	72	179	14.2
CMT_13	183	8.2	61	122	14.6
CMT_14	18	.8	4	10	16.3
CMT_15	78	3.5	18	60	17.8
CMT_16	140	6.2	48	92	14.2
CMT_17	68	3.0	33	35	11.9
CMT_18	67	3.0	32	35	14.0
CMT_19	25	1.1	13	12	20.2
CMT_20	26	1.2	16	20	17.8
CMT_21	34	1.5	17	17	17.7
Unclassed	11	.5	4	7	13.2
Total	2249	100.0	758	1491	14.3

**Table 3:** A summary table of template features

Template	Criterion	Template	Criterion
CMT_1	·Skeletal Class I ·Average angle ·Li-E line and Ls-E line are less than 1 mm, or the sum of the two is less than 2 mm	CMT_12	·Skeletal Class II ·Average and low angle ·Li-E line and Ls-E line are greater than 2.5 mm, or the sum of the two is equal or greater than 4 mm ·Overjet is less than 8 mm
CMT_2	·Skeletal Class I ·High angle ·Li-E line and Ls-E line are less than 1 mm, or the sum of the two is less than 2 mm	CMT_13	·Skeletal Class II ·High angle ·Li-E line and Ls-E line are greater than 2.5 mm, or the sum of the two is equal or greater than 4 mm ·Overjet is less than 8 mm
CMT_3	·Skeletal Class I ·Low angle ·Li-E line and Ls-E line are less than 1 mm, or the sum of the two is less than 2 mm	CMT_14	·Skeletal Class II ·Average and low angle ·Upper incisors linguoclination
CMT_4	·Skeletal Class I ·Average and low angle ·Li-E line and Ls-E line are greater than 1 mm, or the sum of the two is greater than 2 mm	CMT_15	·Skeletal Class II ·High angle ·Open-bite
CMT_5	·Skeletal Class I ·High angle ·Li-E line and Ls-E line are greater than 1 mm, or the sum of the two is greater than 2 mm	CMT_16	·Skeletal Class III ·Average and low angle ·Overjet is less than 0 mm and greater than −3 mm(if the case is low angle, the range of overjet is less than 0 mm and greater than −2 mm)
CMT_6	·Skeletal Class I ·Deep overjet (overjet is equal or greater than 8 mm)	CMT_17	·Skeletal Class III ·High angle ·Overjet is less than 0 mm and greater than − 3 mm
CMT_7	·Skeletal Class II ·Average and low angle ·Li-E line and Ls-E line are less than 2.5 mm, or the sum of the two is less than 4 mm	CMT_18	·Skeletal Class III ·Average angle ·Overjet is less than − 3 mm
CMT_8	·Skeletal Class II ·High angle ·Li-E line and Ls-E line are less than 2.5 mm, or the sum of the two is less than 4 mm	CMT_19	·Skeletal Class III ·High angle ·Overjet is less than − 3 mm
CMT_9	·Skeletal Class II ·Average angle ·Deep overjet (overjet is equal or greater than 8 mm)	CMT_20	·Skeletal Class III ·Low angle ·Overjet is less than −2 mm
CMT_10	·Skeletal Class II ·High angle ·Deep overjet (overjet is equal or greater than 8 mm)	CMT_21	·Skeletal Class III ·High angle ·Open-bite□
CMT_11	·Skeletal Class II ·Low angle ·Deep overjet (overjet is equal or greater than 8 mm)		

### Discriminant analysis

The discriminant accuracy of the total sample was 89.1%, while the cross-validation accuracy was 85.0%, indicating that the clusters were robust. This suggested that the CMTs represented the characteristics of most of the subjects in our research. We entered a total of 34 variables into the discriminant equations (Table S1), and 21 discriminant equations were formed (see supplemental files).

### Treatment evaluation of three clinically typical subclasses

We selected three dentofacial deformity subclasses that are typically observed in clinical practice, and evaluated the treatment outcomes for each of these subclasses. Because the patients in each subclass had received different treatments, we could determine the average changes in a certain subclass after a certain treatment method. The findings of these evaluations can be used not only to assess treatment methods but also to predict treatment outcomes.

#### Example 1:

**Fig. 3:**
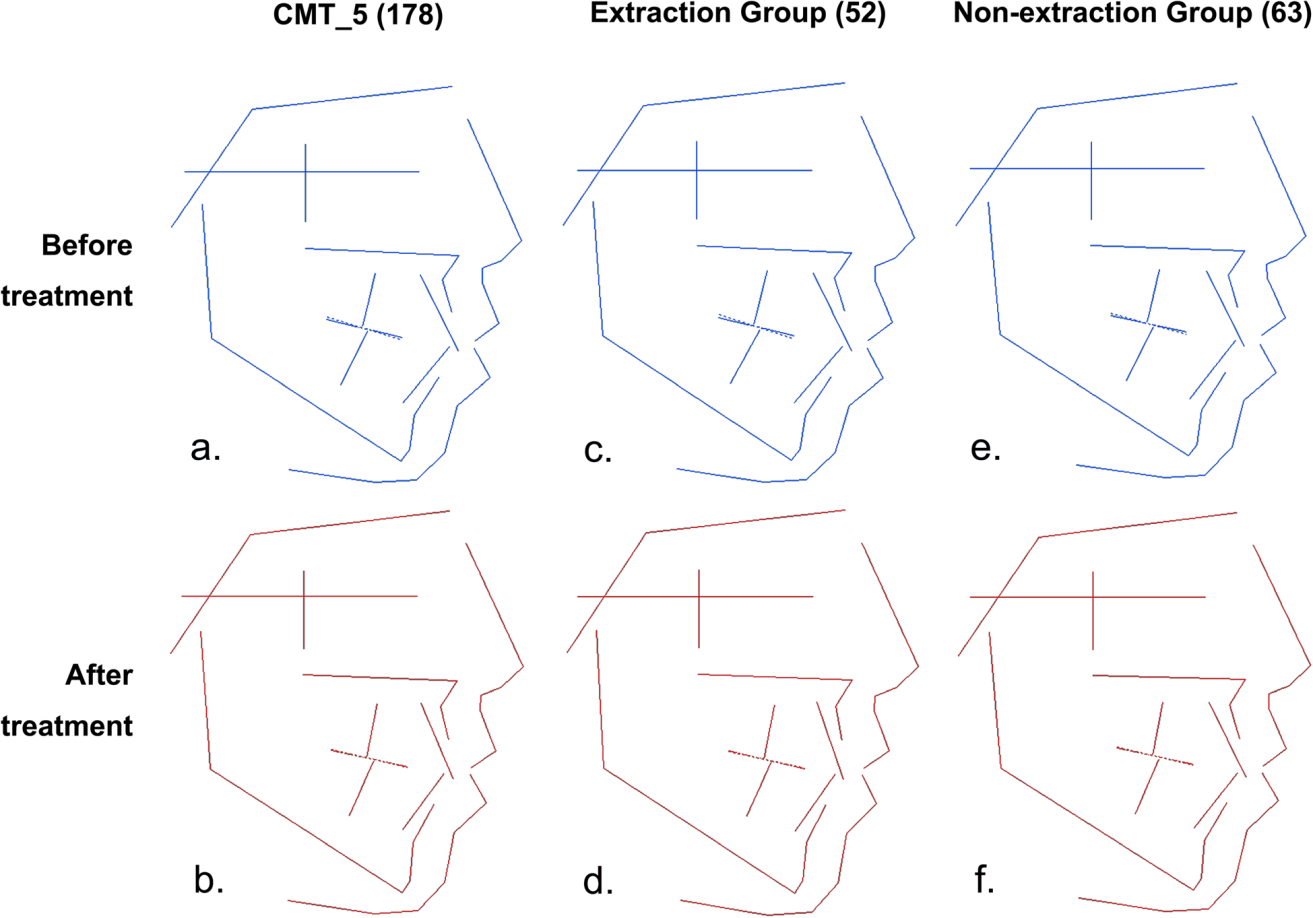
Treatment changes of samples with skeletal class I, high angle and bimaxillary protrusion (CMT_5). a) The average features of CMT_5 before treatment; b) The average features of CMT_5 after treatment; c) The average features of CMT_5 treated with first premolars extraction before treatment; d) The average features of CMT_5 treated with first premolars extraction after treatment; e) The average features of CMT_5 treated with non-extraction before treatment; f) The average features of CMT_5 treated with non-extraction after treatment

CMT_5 represented the average features of patients with skeletal, class I, high angle, bimaxillary protrusion. Figure 3 shows the average changes in patients in the CMT_5 group before and after orthodontic treatment (Fig. 3a, b) as well as the morphology diagrams after treatment with (Fig. 3c, d) or without (Fig. 3e, f) extraction of the first premolars. The treatment results in this subclass were as follows: (1) In first premolar extraction group, the forward inclination and protrusion of both the upper and lower incisors decreased significantly, and the soft tissues, upper and lower lips, and profile improved significantly. (2) In the group treated without extraction of the first premolars, there were no significant improvements in the inclination of the incisors, protrusion of the lips and the profile after treatment. (3) Thus, in patients with craniofacial features of skeletal, class I, high-angle, bimaxillary protrusion, the extraction method is a better alternative that results in better treatment efficacy.

#### Example 2:

**Fig. 4:**
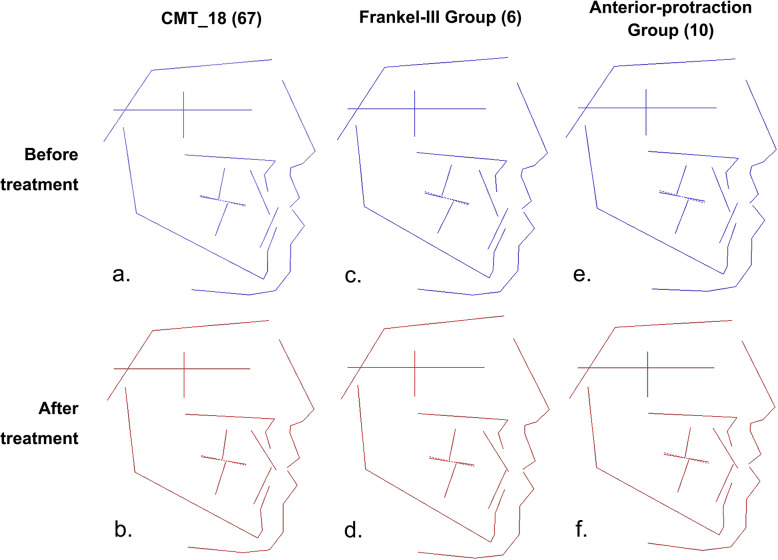
Treatment changes of samples with skeletal class III, average angle, retruded maxilla and protruded mandible (CMT_18). a) The average features of CMT_18 before treatment; b) The average features of CMT_18 after treatment; c) The average features of CMT_18 treated with Frankel III appliance before treatment; d) The average features of CMT_18 treated with Frankel III appliance after treatment; e) The average features of CMT_18 treated with anterior protraction before treatment; f) The average features of CMT_18 treated with anterior protraction after treatment

CMT_18 represented the average features of patients with skeletal, class III, average angle, maxillary retrusion, and mandibular protrusion. Figure 4 shows the average changes in patients in the CMT_18 group before and after orthodontic treatment (Fig. 4a, b) as well as the morphology diagrams after treatment with the Frankel III appliance (F-III; Fig. 4c, d) or with anterior protraction (Fig. 4e, f) without extraction. The treatment results in this subclass were as follows: (1) There were significant changes in the jaw relationships in both groups, but the changes were more obvious in the anterior protraction group. (2) The mandibular plane angle increased and the profile obviously improved in both groups. (3) In patients with craniofacial features of skeletal, class III, average angle, maxillary retrusion, and mandibular protrusion, the F-III functional appliance and anterior protraction were both effective. (4) Compared with the F-III functional appliance, anterior protraction had greater efficacy, which is consistent with previous studies.

#### Example 3:

**Fig. 5:**
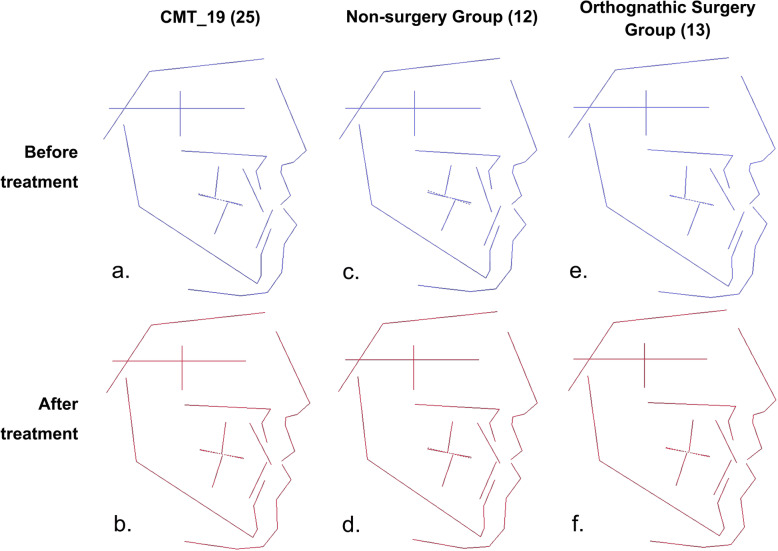
Treatment changes of samples with skeletal class III, high angle, retruded maxilla and protruded mandible (CMT_19). a) The average features of CMT_19 before treatment; b) The average features of CMT_19 after treatment; c) The average features of CMT_19 treated with non-surgery before treatment; d) The average features of CMT_19 treated with non-surgery after treatment; e) The average features of CMT_19 treated with orthognathic surgery before treatment; f) The average features of CMT_19 treated with orthognathic surgery after treatment

CMT_19 represented the average features of patients with skeletal, class III, high angle, maxillary retrusion, and mandibular protrusion. Figure 5 shows the average changes in patients in the CMT_19 group before and after treatment (Fig. 5a, b) as well as the morphology diagrams after non-surgical treatment (Fig. 5c, d) or after orthognathic surgery (Fig. 5e, f). The results were as follows: (1) Orthognathic surgery could improve jaw relationships and coordinate the hard and soft tissues more effectively. (2) Non-surgical treatment could change certain craniofacial features to a limited extent, but with less obvious effects than orthognathic surgery.

## Discussion

The main purpose of this study was to form biologically informative, digital templates of dentofacial deformities, so as to enable rapid, computerized, automatic diagnoses by matching the imaging data of a new patient with the characteristic templates. A comprehensive and accurate classification is critical to the process of template establishment. Subclasses of dentofacial deformities have been described in the literature [20, 23, 25]. However, most such classification systems rely on a single angle measurement or molar relationship. A comprehensive classification of deformity patterns requires subclasses that are not based on a single variable alone. Moyers et al. classified 697 patients with class II malocclusions into six horizontal and five vertical divisions by using cluster analysis, and developed diagnostic definitions and designed treatment plans pertaining to each subclass [20].

In this study, we used cluster analyses with no a priori definitions to establish a classification system of craniofacial deformities. This research was carefully designed to avoid the shortcomings that were limited by sample size or selection bias. We included a sample of 2249 patients with any type of malocclusion and performed a comprehensive analysis of as many cephalometric landmarks as possible. Considering the ethnic diversity, we only recruited Han population into the sample. The cephalometric measurements were obtained by working out the average locations of the selected landmarks. All the landmarks were independently identified and located by three professionally trained orthodontic residents. The data were calibrated, which helped to ensure the accuracy of the results. A total of 21 CMTs were established representing almost all patterns of dentofacial deformities. These cephalometric values and CMTs could not completely represent of an ethnicity, but can still be used for reference.

The most commonly used method to evaluate therapeutic effects in previous studies was choosing one type of malocclusion (mostly based on Angle’s classification), and then statistically analyzing cephalometric measurements before and after treatment. This method is simple, appropriate to understand and widely used. However, there are two obvious flaws in this method. First, the classification of the entire sample is based on a single feature, such as molar relationships or jaw relationships, ignoring the influences of other confounding features. Second, line and angle measurements cannot reflect the overall dentofacial morphology, unlike the main landmarks shown on CMTs (Fig. 1). The CMTs formed from this cluster analysis are based on landmark information, and therefore, the resultant classification based on these CMTs will ensure a high degree of sample similarity within the same subclass. The CMT of a large sample represents the mean morphology of a pattern of similar structures, and the changes in the CMT before and after treatment represent the mean morphological changes in samples with similar structures. This close similarity will greatly increase the reliability of efficacy analysis. CMTs contains much more cranial and maxillofacial deformities than traditional method.

Another obvious advantage of CMTs is that they can be calculated on the computer and display the changes in the position of landmarks. Therefore, the changes before and after treatment can be seen intuitively, which is more conducive to orthodontists’ judgment and doctor–patient communication. Although hand-traced templates have been used to evaluate the dentofacial characteristics of patients [5, 6], predict growth [7] and perform diagnoses [8], few such templates have been used for treatment evaluation. CMT analysis is akin to traditional superimposition analysis, but is more effective than superimposition and better reflects average changes for a class of samples.

For example, we subdivided patients in the CMT_5 subclass, which is a common type in clinical practice, into two groups based on whether or not they underwent tooth extraction. Great improvement in the profile was seen in the extraction group, with significant reductions in the inclination of the upper and lower incisors and in lip protrusion (Fig. 3). In contrast, the soft and hard tissues were not well improved in the non-extraction group, and protrusive incisors and a convex profile persisted after treatment.

The clinical effects of functional appliances are controversial, whereas anterior protraction treatment for skeletal, class III malocclusion is commonly viewed as being efficient [26]. We therefore compared the efficacy of the F-III appliance and anterior protraction in the patients in the CMT_18 subclass, which pertained to skeletal, class III malocclusions (Fig. 4). We found that the ANB angle was more significantly improved in the anterior protraction group than in the F-III functional appliance group, which is consistent with previous studies. Anterior protraction seemed to be more beneficial for skeletal improvement, whereas the F-III appliance seemed to better improve soft tissues. The profile improved well in both groups, without obvious differences. The results seemed to suggest that both methods could be used to treat patients with skeletal, class III malocclusions in mixed dentition or early permanent dentition.

Since the computer system is not yet operational and needs further improvement, there are two main ways to use templates today. First, these templates are a morphological template based on coordinate clustering. Morphological matching could be used quickly in the clinic for reference by these templates, but it is not the most accurate way to use. Second, to get an accurate result, a computer is needed to substitute the coordinate value into the equation. Substitute the coordinate values of the sample into the 21 discriminant equations to see which equation results in a higher value. The sample corresponds to the group with the largest result value of the discriminant equation.

However, the best way to do this is to automate it all. Therefore, the goal in the future is to use software to achieve accurate discrimination. Although we only partially contrasted the effects of several treatment methods in a relatively large number of samples, this is exploratory investigation can provide a basis for future research into the prediction of treatment outcomes in patients with dentofacial deformities. New cases will be gradually accumulated in the informative CMT database, which will be enriched with information about the outcomes of different treatment methods.

In addition to treatment evaluation, this study is superior in many aspects, including the large sample size, cross-validation, and general diversity. Most importantly, a priori definitions were not applied before clustering. In this study, all 2249 patients without a priori pattern definitions were divided by cluster analysis based on the coordinates of their lateral cephalograms rather than the conventional Angle’s classification. In this way, samples of each subclass had integral dentofacial similarity, and represented almost all types of dentofacial deformities.

## Conclusion

This study demonstrates the utility of clustering methods for grouping dentofacial deformities with similar features in the age of big data. We established 21 biologically informative templates of different dentofacial deformities. Our study will provide references for rapid template matching and thereby enable the fast, computerized diagnosis of dentofacial deformities. In addition, comparative research on treatment efficacies in samples with highly similar dentofacial morphologies based on the CMTs will be more objective. The CMTs formed in this study will enable rapid diagnoses, the designing of treatment plans based on the evaluation of the treatment outcomes of samples affiliated with the same CMT, and the prediction of treatment outcomes. The findings of this study are necessary to develop novel, biological data-driven methods for dentofacial diagnosis and treatment outcome prediction. The CMTs described in this study are expected to enable the critical transition to computerized, fully automatic, pinpoint measurements in the near future.

## Supplementary Information

**Additional file 1: Table S1.** There are 34 coordinates of the landmarks entering into the discriminant equations. Each of them are represented as variables Xn in the equations.

## Data Availability

The datasets used and/or analyzed during the current study are available from the corresponding author on reasonable request.
