# Supplementary material for: Development of digital diagnostic templates by cluster analysis based on 2249 lateral cephalograms of Chinese Han population
Source: Head Face Med. 2022 Feb 14;18:5. doi: 10.1186/s13005-022-00309-2 (PMC8842905; doi:10.1186/s13005-022-00309-2)
Supplement: Supplementary file 1 — Additional file 1: Table S1. There are 34 coordinates of the landmarks entering into the discriminant equations. Each of them are represented as variables Xn in the equations. [file 13005_2022_309_MOESM1_ESM.doc]

Table S1: There are 34 coordinates of the landmarks entering into the discriminant equations. Each of them are represented as variables Xn in the equations.

| Name of variables | Coordinates of the landmarks |
| --- | --- |
| X1 | A.x |
| X2 | Pr(P).x |
| X3 | UIE.x |
| X4 | UIE.y |
| X5 | UIA.x |
| X6 | UIR.x |
| X7 | UIR.y |
| X8 | UMC.y |
| X9 | LIE.x |
| X10 | LIE.y |
| X11 | Id.x |
| X12 | LIA.x |
| X13 | LIA.y |
| X14 | LIR.x |
| X15 | LIR.y |
| X16 | B.x |
| X17 | B.y |
| X18 | Pg.y |
| X19 | Gn.x |
| X20 | Gn.y |
| X21 | Tgo.y |
| X22 | GoP.x |
| X23 | Interdental(M).y |
| X24 | Ns.x |
| X25 | Prn.x |
| X26 | UL.x |
| X27 | Stoms.x |
| X28 | Stomi.x |
| X29 | Stomi.y |
| X30 | Pos.x |
| X31 | Pos.y |
| X32 | LRp.x |
| X33 | LRp.y_a |
| X34 | LRa.x |

.x: the horizontal coordinate of the landmark;

.y: the vertical coordinate of the landmark.

Supplemental instruction: All the discriminant equations of the CMTs are showed as follows. Equation Y1~Y21 are representing relative equation CMT_1~21.

**Y1**=6.629X1-6.413X2+2.136X3-3.459X4-1.956X5+1.58X6+0.846X7+0.303X8+3.771X9+3.817X10-0.282X11+2.418X12-4.631X13-7.700X14-6.578X15-1.664X16-0.198X17-1.315X18+2.185X19+4.535X20+0.820X21-1.509X22+2.088X23+4.845X24-1.344X25-2.187X26+2.448X27+0.469X28+0.890X29+0.335X30-1.401X31-2.154X32+1.571X33+2.511X34-407.610

**Y2**=6.0311.749X1-5.467X2+1.500X3-3.225X4-2.672X5+1.505X6+0.751X7+0.724X8+3.483X9+3.621X10+0.268X11+2.130X12-4.592X13-7.150X14-6.397X15-1.478X16-0.879X17-0.350X18+1.741X19+3.421X20+0.997X21-1.175X22+2.261X23+5.143X24-1.458X25-1.987X26+2.523X27+0.424X28+0.667X29+0.501X30-1.340X31-2.378X32+1.618X33+2.614X34-409.970

**Y3**=6.770X1-5.971X2+1.914X3-3.406X4-1.746X5+0.529X6+0.218X7+0.221X8+3.421X9+3.745X10-1.134X11+2.650X12-4.910X13-6.685X14-6.014X15-2.153X16-0.450X17+0.426X18+2.479X19+3.666X20+0.336X21-1.872X22+2.130X23+4.944X24-1.385X25-2.184X26+3.505X27+0.278X28+0.775X29+0.474X30-1.325X31-2.084X32+1.729X33+2.339X34-417.653

**Y4**=6.770X1-5.731X2+1.911X3-3.579X4-2.295X5+1.267X6+0.677X7+0.367X8+4.004X9+3.529X10-1.158X11+1.473X12-4.545X13-7.697X14-6.383X15-1.154X16-1.143X17-0.303X18+2.118X19+3.885X20+0.502X21-1.501X22+2.348X23+4.985X24-1.655X25-1.657X26+2.853X27+0.495X28+0.767X29+0.071X30-1.233X31-1.063X32+2.371X33+2.821X34-401.999

**Y5**=5.853X1-6.343X2+2.334X3-3.452X4-1.712X5+1.211X6+0.624X7+0.428X8+3.862X9+3.321X10-0.316X11+2.250X12-4.052X13-7.945X14-6.570X15-0.924X16-0.622X17-0.658X18+1.402X19+3.522X20+0.831X21-1.238X22+2.291X23+5.012X24-1.834X25-1.497X26+2.335X27+0.615X28+0.974X29+-0.130X30-1.351X31-1.720X32+1.889X33+2.821X34-402.835

**Y6**=6.289X1-5.661X2+4.413X3-2.917X4-0.977X5-0.480X6-0.599X7+0.616X8+2.734X9+4.408X10-1.369X11+1.968X12-4.759X13-7.015X14-6.357X15-1.726X16-0.379X17-0.615X18+2.397X19+3.984X20+0.730X21-1.494X22+2.257X23+4.814X24-1.324X25-2.173X26+2.944X27+0.043X28+0.355X29+0.430X30-1.389X31-2.293X32+1.884X33+2.656X34-409.770

**Y7**=7.311X1-5.337X2+1.686X3-3.296X4-1.009X5-0.139X6-0.248X7+0.407X8+3.786X9+4.299X10-0.735X11+1.400X12-3.735X13-8.136X14-6.696X15-1.811X16-1.012X17-0.246X18+2.684X19+3.183X20+0.655X21-1.559X22+2.283X23+4.510X24-1.171X25-2.411X26+3.191X27+0.261X28+0.721X29+0.333X30-1.202X31-1.582X32+2.247X33+2.920X34-403.615

**Y8**=6.779X1-5.420X2+2.054X3-3.823X4-1.946X5+1.213X6+0.612X7+0.090X8+4.060X9+3.804X10-0.540X11+1.920X12-4.385X13-7.662X14-6.259X15-2.532X16-0.748X17-0.333X18+2.625X19+3.159X20+1.028X21-1.290X22+2.976X23+4.326X24-0.936X25-2.213X26+2.190X27+0.559X28+0.908X29+0.270X30-1.128X31-1.379X32+1.544X33+2.017X34-396.532

**Y9**=6.670X1-5.029X2+3.922X3-3.524X4-1.066X5-0.578X6-0.612X7+0.456X8+3.894X9+4.318X10-1.717X11+2.090X12-4.318X13-8.048X14-6.527X15-2.120X16-0.733X17-0.852X18+2.571X19+3.666X20+0.589X21-1.463X22+3.199X23+4.447X24-1.352X25-2.093X26+5.543X27-2.834X28+0.746X29+0.199X30-1.011X31-2.658X32+1.993X33+3.787X34-410.721

**Y10**=6.947X1-5.184X2+3.721X3-4.126X4-1.657X5+0.225X6-0.027X7-0.547X8+2.869X9+3.281X10-1.318X11+2.265X12-4.356X13-7.776X14-6.437X15-2.520X16-0.155X17-0.290X18+2.077X19+3.054X20+0.749X21-1.084X22+4.134X23+4.496X24-1.462X25-2.152X26+3.057X27+0.077X28+0.498X29+0.187X30-1.027X31-1.867X32+2.311X33+3.408X34-408.389

**Y11**=7.409X1-5.547X2+3.878X3-3.199X4-1.200X5-0.743X6-0.681X7+1.045X8+3.489X9+4.169X10-2.203X11+1.417X12-4.510X13-5.874X14-5.646X15-1.898X16-0.750X17+0.454X18+2.544X19+2.618X20+0.330X21-1.603X22+2.177X23+4.366X24-1.372X25-2.315X26+3.062X27+0.359X28+0.359X29+0.315X30-0.998X31-3.219X32+1.827X33+3.132X34-422.943

**Y12**=6.486X1-5.532X2+2.825X3-3.921X4-1.507X5+0.705X6+0.321X7+0.593X8+4.342X9+3.601X10-1.792X11+1.885X12-3.877X13-7.212X14-5.974X15-2.057X16-0.999X17-0.303X18+2.662X19+3.442X20+0.624X21-1.560X22+2.544X23+4.452X24-1.513X25-1.869X26+2.687X27+0.305X28+0.786X29+0.048X30-1.203X31-2.429X32+1.709X33+3.153X34-401.258

**Y13**=6.798X1-6.111X2+2.884X3-3.957X4-1.309X5+0.425X6+0.115X7+1.043X8+3.868X9+3.045X10-0.872X11+1.549X12-4.690X13-7.043X14-5.709X15-2.116X16+0.131X17-0.968X18+2.053X19+3.543X20+0.913X21-1.312X22+2.078X23+4.456X24-1.434X25-1.882X26+2.288X27+0.721X28+0.933X29-0.089X30-1.181X31-1.072X32+1.836X33+2.345X34-409.394

**Y14**=7.922X1-2.867X2+1.389X3-1.278X4+4.942X5-10.782X6-2.122X7-0.618X8+4.830X9+4.364X10-0.771X11+2.357X12-5.467X13-8.290X14-2.358X15-1.809X16-0.448X17+0.391X18+2.134X19+2.205X20+0.583X21-1.308X22+2.767X23+4.704X24-1.508X25-2.953X26+5.081X27+1.282X28-0.161X29-0.173X30-0.543X31-0.848X32-0.327X33+1.168X34-470.037

**Y15**=7.225X1-5.096X2+3.250X3-2.701X4-1.813X5+1.045X6+0.541X7-0.712X8+1.972X9+1.903X10-0.600X11+2.970X12-4.884X13-8.138X14-6.712X15-2.937X16+0.206X17-0.162X18+1.669X19+3.096X20+0.931X21-1.033X22+3.817X23+4.474X24-1.480X25-2.138X26+2.886X27+0.660X28+0.852X29+0.019X30-1.309X31-1.404X32+2.160X33+2.765X34-418.338

**Y16**=5.592X1-5.758X2+0.019X3-2.827X4-2.416X5+0.744X6+0.327X7+1.026X8+5.433X9+4.108X10-0.927X11+2.546X12-4.653X13-7.818X14-6.806X15-0.327X16-1.637X17+0.503X18+1.249X19+3.292X20+0.677X21-1.397X22+1.610X23+5.395X24-1.375X25-2.507X26+3.785X27+0.336X28+0.641X29+0.324X30-1.347X31-2.082X32+2.392X33+3.334X34-410.255

**Y17**=5.845X1-5.693X2-0.995X3-3.071X4-2.320X5+0.304X6+0.060X7+1.577X8+7.203X9+5.104X10-0.533X11+2.328X12-4.334X13-9.302X14-7.828X15-0.980X16-1.937X17+0.721X18+1.354X19+2.696X20+0.893X21-1.104X22+1.394X23+5.162X24-1.373X25-2.787X26+4.183X27+0.506X28+0.558X29+0.380X30-1.355X31-1.475X32+2.926X33+3.377X34-410.320

**Y18**=5.409X1-6.629X2-1.853X3-4.234X4-2.554X5-0.317X6-0.308X7+1.646X8+8.327X9+6.397X10-0.655X11+2.923X12-4.005X13-8.683X14-7.489X15-0.042X16-2.167X17+1.628X18+0.758X19+2.275X20+0.727X21-1.132X22+0.802X23+5.587X24-1.387X25-3.378X26+5.646X27+0.244X28+0.655X29+0.562X30-1.537X31-1.732X32+2.816X33+3.041X34-432.777

**Y19**=4.421X1-5.234X2-2.641X3-3.994X4-3.855X5+1.184X6+0.579X7+3.534X8+8.339X9+5.100X10-1.687X11+3.087X12-4.268X13-8.972X14-7.692X15+0.183X16-2.225X17+1.508X18+0.948X19+1.294X20+0.976X21-0.821X22-0.361X23+6.079X24-1.576X25-2.503X26+4.116X27+0.419X28+0.848X29+0.203X30-1.266X31-2.088X32+3.220X33+4.691X34-464.203

**Y20**=5.042X1-7.050X2-1.827X3-4.649X4-3.460X5+0.710X6+0.365X7+2.117X8+7.938X9+6.270X10-1.443X11+3.589X12-3.963X13-9.209X14-8.357X15+0.206X16-1.881X17+2.608X18+0.575X19+2.006X20+0.081X21-1.211X22-0.250X23+5.788X24-1.339X25-3.085X26+5.185X27+0.483X28+0.274X29+0.623X30-1.519X31-0.966X32+3.817X33+3.570X34-445.359

**Y21**=5.480X1-4.568X2+0.118X3-2.600X4-3.130X5+1.668X6+0.904X7+7.119X8+4.784X9+3.160X10-1.117X11+2.922X12-4.246X13-2.890X14-3.235X15-0.097X16-2.234X17+1.193X18+1.123X19+1.320X20+1.176X21-0.927X22-3.604X23+5.378X24-1.426X25-1.231X26+1.952X27+0.124X28+1.241X29+0.288X30-1.149X31-5.115X32-1.619X33+0.861X34-450.067
